# The benefits and risks of pembrolizumab in combination with chemotherapy as first-line therapy in small-cell lung cancer: a single-arm meta-analysis of noncomparative clinical studies and randomized control trials

**DOI:** 10.1186/s12957-021-02410-3

**Published:** 2021-10-14

**Authors:** Qiangyun Liu, Yixuan Zhang, Miaowen Liu, Ruoxin Xu, Fengming Yi, Yiping Wei, Shuqiang Zhu, Wenxiong Zhang

**Affiliations:** 1 Department of Thoracic Surgery, The Second Affiliated Hospital of Nanchang University, 1 Minde Road, Nanchang, 330006 China; 2 Jiangxi Medical College, Nanchang University, Nanchang, 330006 China; 3 Department of Oncology, The Second Affiliated Hospital of Nanchang University, 1 Minde Road, Nanchang, 330006 China; 4 Department of Cardiovascular Surgery, The Second Affiliated Hospital of Nanchang University, Nanchang, 330006 China

**Keywords:** Pembrolizumab, Chemotherapy, Small-cell lung cancer, Meta-analysis, Systematic review

## Abstract

**Background:**

Although pembrolizumab has shown clinical benefit in patients with small-cell lung cancer (SCLC), its actual efficacy in combination with a conventional chemotherapy drug has not been determined. We performed this study to discern the efficacy and risk of pembrolizumab in combination with chemotherapy as first-line therapy in SCLC patients.

**Methods:**

We systematically searched the PubMed, ScienceDirect, Cochrane Library, Scopus, Ovid MEDLINE, Embase, Web of Science, and Google Scholar databases for relevant studies. The main outcomes were overall survival (OS) and progression-free survival (PFS).

**Results:**

We identified 2980 articles and included 6 studies (5 were noncomparative open-label studies and 1 was a randomized controlled trial [RCT]) involving 396 patients in our meta-analysis. The pooled median OS (mOS) was 9.6 months (95% CI, 8.0-11.2), and the pooled median PFS (mPFS) was 4.2 months (95% CI, 2.2-6.1). The 1-year overall survival rate (OSR-1y) and 6-month progression-free survival rate (PFSR-6m) were 45.1% (95% CI, 33-57.2%) and 41.6% (95% CI, 24.3-59%), respectively. The objective response rate (ORR) was 38.8% (95% CI, 11.9-65.67%), disease control rate (DCR) was 69.30% (95% CI, 51.6-87.0%), complete response (CR) was 2.20% (95% CI, 0.8-3.7%), partial response (PR) was 34.70% (95% CI, 7.8-61.5%), and stable disease (SD) was 20.90% (95% CI, 9.1-32.6%). The grade 3-4 adverse effect (AE) rate was 20.88% (95% CI, 1.22-54.85%). The most common AEs were neutropenia (90.16%), anemia (53.21%), dysphagia (41.96%), platelet count decrease (34.87%), and esophagitis (32.89%); severe AEs included neutropenia, respiratory failure, pneumonitis, acute coronary syndrome, and colitis/intestinal ischemia.

**Conclusions:**

The combination of pembrolizumab with conventional chemotherapy is an effective therapeutic schedule with acceptable and manageable efficacy and toxicity in patients with SCLC. More high-quality and well-designed RCTs with large sample sizes are warranted to further validate our findings.

**Supplementary Information:**

The online version contains supplementary material available at 10.1186/s12957-021-02410-3.

## Novelty and impact statements

This meta-analysis offers the most comprehensive and latest evidence for the management of small-cell lung cancer. The results suggested that pembrolizumab with conventional chemotherapy is a beneficial therapeutic schedule with assessable PFS, OS, ORR, and grade 3-4 AEs. Subgroup analyses indicated that patient age (< 65 years old) and SCLC phase (LS-SCLC) are the main factors affecting efficacy of pembrolizumab plus chemotherapy.

## Background

As a type of invasive tumor with few treatment options and undesirable prognosis [1], small-cell lung cancer (SCLC) makes up a relatively significant proportion (18-20%) of all new lung cancer cases, 80-85% of which are diagnosed with extensive-stage disease worldwide [2]. Platinum-based chemotherapy represents the foundation of treatment for patients with SCLC, but not much progress has been made in related therapeutic drugs [3]. Although SCLC is initially sensitive to chemotherapy and radiation, it always relapses within 6 months with a low 5-year survival rate (5%) [4–6], and the emergence of drug resistance is still a considerable clinical challenge [7]. In recent years, immunotherapy has been recommended for the treatment of SCLC and has shown relatively good efficacy and safety.

As one of the earliest researched and developed immunotherapy drugs, pembrolizumab was approved for treating SCLC in 2019 [8]; moreover, the efficacy of pembrolizumab plus chemotherapy in NSCLC was also demonstrated that substantially improved OS and PFS [9]; however, the efficacy and risk of pembrolizumab plus conventional chemotherapy have yet to be determined [10, 11]. Charles et al. [12] demonstrated that the combination of pembrolizumab and a chemotherapy drug had better efficacy than the chemotherapy drug alone (hazard ratio [HR] of progression-free survival (PFS): 0.80; 95% CI, 0.64-0.98; *p* = 0.0164). Multiple noncomparative studies [13–17] have confirmed its acceptable efficacy with a median overall survival (mOS) of 9.7-10.8 months. To better guide clinical treatment, we performed this study to comprehensively analyze the benefit and risk of pembrolizumab plus chemotherapy.

## Materials and methods

We conducted this meta-analysis following the Preferred Reporting Items for Systematic Review and Meta-Analysis (PRISMA) guidelines (Table S1) (PROSPERO registration: CRD42020218612).

### Search strategy

We retrieved relevant literature through searches in PubMed, Web of Science, The Cochrane Library, ScienceDirect, Scopus, Ovid MEDLINE, and Embase. The last search was on December 20, 2020. The combined search term with Medical Subject Headings (MeSH) terms applied were “Pembrolizumab,” “Chemotherapy,” and “Small-cell lung cancer” (detailed search strategy in Table S2). The unpublished papers during the study and references of included studies were also searched.

### Selection criteria

Articles meeting the following conditions were included:

1. Participants: Adult patients diagnosed with SCLC, regardless of extensive-stage small-cell lung cancer (ES-SCLC) or limited small-cell lung cancer (LS-SCLC) without restriction and obvious differences in sex, race, region, or nationality
2. Interventions: Treatment arm included pembrolizumab and a chemotherapy drug whether or not a comparison had been implemented
3. Outcomes: The primary study outcomes were OS, PFS, objective response rate (ORR), disease control rate (DCR), and grade 3-4 treatment-related adverse effect rate (grade 3-4 AEs%). All treatment-related AE ranks were evaluated by the Lung Cancer Symptom Scale and Life-5 Dimension questionnaire [18]
4. Study design: Prospective open-label clinical study and randomized control trials (RCTs)

The latest studies from 2017 to 2020 were adopted to extract data of survival outcomes, drug response, and adverse effects (AEs). We excluded meta-analyses, reviews without available original data, human experiments without pembrolizumab + chemotherapy exposure and relevant outcome data, animal experiments, and studies with duplicated data or only abstracts.

### Data extraction

The following data were extracted by two investigators independently: first author, year of publication, registered number, country, number of participants, participant characteristics (age, sex, phase of SCLC, and Eastern Cooperative Oncology Group [ECOG] status), chemotherapy drug, pembrolizumab arm, and study design. We extracted the total relevant data and subgroup characteristics from noncomparative studies and extracted valuable data on therapeutic regimens and control groups from RCTs. Antitumor efficacy indices (survival outcomes including mPFS, mOS, OSR, and PFSR; drug responses including ORR, DCR, complete response [CR], partial response [PR], stable disease [SD], median duration of response [mDOR], and ongoing response) and AEs (total AEs and grade 3-4 AEs) were also extracted. Another investigator resolved any differences, and all original data were recorded in corresponding tables.

### Quality assessment

RCTs were assessed using the Cochrane risk of bias tool and did not identify existing allocation concealment and blinding methods, as well as generated random sequences, provided complete outcome data, and reported no selective outcome without other bias [19]. GRADE quality assessment was also conducted through therapeutic regimens for the survival outcomes, drug response, and AEs to confirm the high quality of the results [20]. The noncomparative studies we included came from top journals or excellent scientists, with high quality and credibility.

### Statistical analysis

We analyzed the survival outcomes and drug response of pembrolizumab combined with a conventional chemotherapy drug. We input total therapeutic regimens for the percentage of survival outcomes and number of participants and calculated the standard errors of these quasinormal distribution “rates” using Stata (version 16.0). We used the “rate” and standard errors to obtain the 95% confidence interval (CI) [21, 22]. Finally, the pooled effect size (ES), which represented the median “rate” with 95% CI, was output [23], and we used the double arcsine method to converse and correct the rate less than 0.2 [24]. We estimated the mean using the values of the median OS and PFS, low and high end of the range, and the sample size to output the pooled ES [25]. The pooled ES was used to estimate the efficacy and risk of pembrolizumab plus chemotherapy. The heterogeneity of the included studies was assessed by the Cochrane Q chi-square test and the *I*^2^ statistic, with 25-50%, 50-75%, and > 75% representing low, moderate, and high heterogeneity, respectively [26]. *P* < 0.1 for the *Q* test was deemed to indicate high heterogeneity. Random-effects models were applied to all pooled ESs because the included studies were mostly single-arm (noncomparative) experiments without control groups and had a tendency toward high heterogeneity [27]. *P* < 0.05 was regarded as statistically significant.

We also performed subgroup analyses of the study design (noncomparative open-label study, randomized control study), chemotherapy drug (platinum plus etoposide; platinum plus etoposide and paclitaxel), clinical setting, SCLC phase (ES-SCLC, LS-SCLC), study phase (I, II, III, I/II), median participant age (≤ 65 years old, > 65 years old), region (America, Korea), and whether the treatment was combined with radiation (Yes, No).

We assessed the publication of the primary outcomes through the Egger’s and Begg’s linear regression tests [28]. We used Stata 16.0 (StataCorp, USA, http://www.stata.com) to conduct all statistical analyses.

## Results

### Search results and study quality assessment

**Fig. 1:**
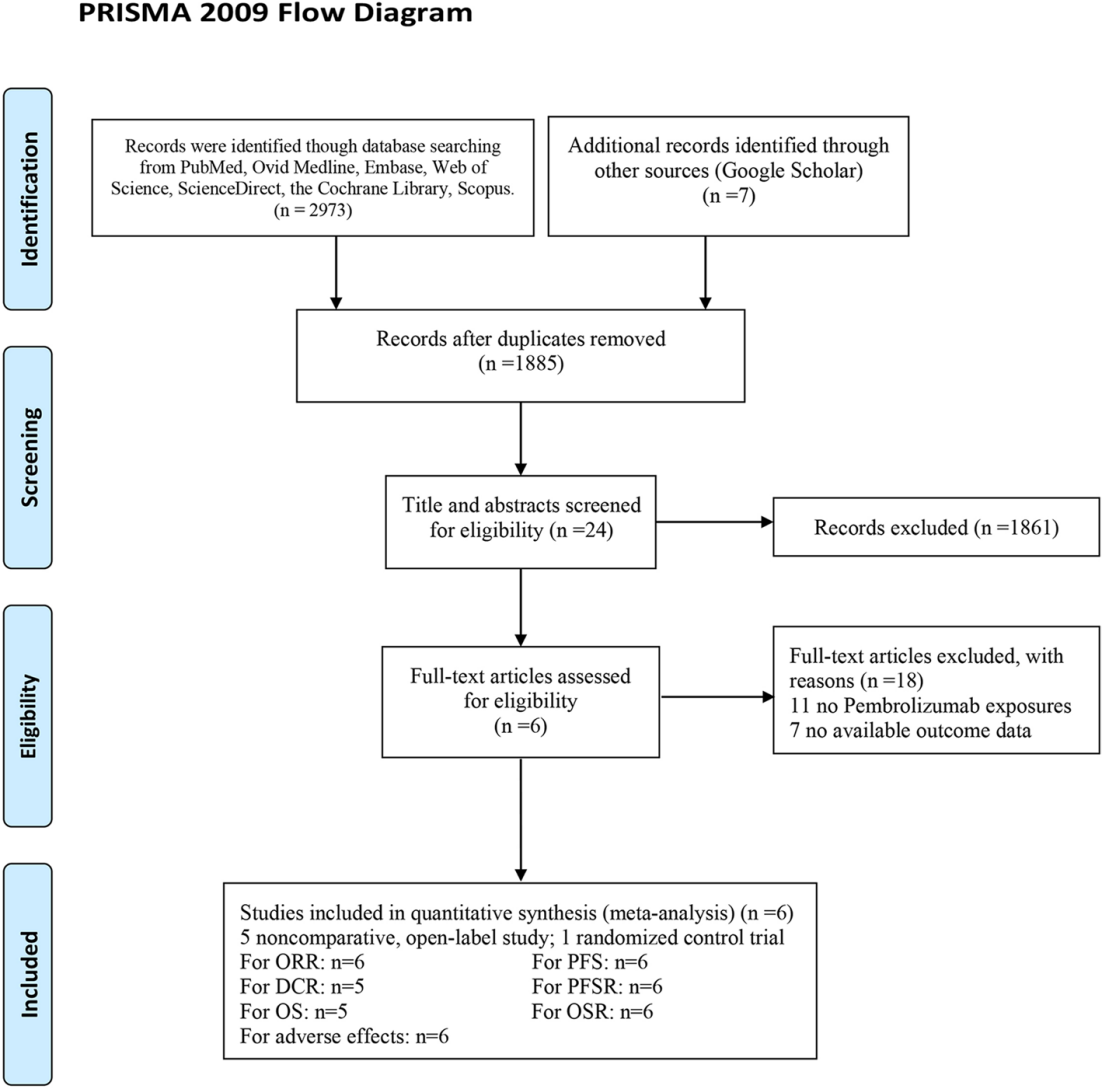
Flow chart of study selection

We initially identified 2980 studies. Finally, 6 studies involving 396 patients were included in our study (Fig. 1) [12–17]. Of the 6 studies, 1 was a randomized controlled trial (RCT) [11] and 5 were noncomparative prospective open-label clinical studies [12–16]. Welsh et al. [15] and Heymach et al. [17] came from the same clinical study but with different participants (38 ES-SCLC patients and 40 LS-SCLC patients, respectively) not involving duplication.

The RCT [12] was of high quality (scored 5 points), and the noncomparative studies [13–17] we included came from top journals or excellent researchers or scholars, which guaranteed their high quality and reliability. The baseline characteristics and primary evaluating indicators of the included studies are summarized in Tables 1 and 2. According to the GRADE method, all of the results were of medium-high quality (Table S3).

**Table 1:** Characteristics of included studies

Study		Register number	Country	Patients	Age (years)	Male (%)	Phase	ECOG status	Chemotherapy drug	Pembrolizumab arm	Study design
2020	Charles [12]	NCT03066778	America	Arm A (*n* = 228)	64 (24-81)	66.7	ES-SCLC	0/1	Platinum plus etoposide	Pembrolizumab 200 mg, Q3W, iv for 35 cycles	Randomized controlled trial
				Arm B (*n* = 225)	65 (37-83)	63.1		0/1		-	
2017	Ott [13]	NCT02054806	America	24	60.5 (41-80)	58.3	ES-SCLC	0/1	Platinum plus etoposide	Pembrolizumab 10 mg/kg, Q2W, iv for 24 months	Noncomparative open-label study
2018	Shirish [14]	NCT02359019	America	45	66 (50-86)	56	ES-SCLC	0/1	Platinum plus etoposide	Pembrolizumab 200 mg, Q3W, iv for 24 months	Noncomparative open-label study
2019	Kim [15]	NCT02551432	Korea	26	68.5 (54–78)	88.5	ES-SCLC	0/1	Platinum plus etoposide and paclitaxel	Pembrolizumab 175 mg/m2, Q3W, iv for 5 cycles	Noncomparative open-label study
2019	Welsh [16]	NCT02402920	America	38	65 (37–79)	61	ES-SCLC	-	Platinum plus etoposide	Pembrolizumab 200 mg, Q3W, iv for 16 cycles	Noncomparative open-label study
2020	Welsh [17]	NCT02402920	America	40	64 (41−79)	40	LS-SCLC	-	Platinum plus etoposide	Pembrolizumab 200 mg, Q3W, iv for up to 16 cycles	Noncomparative open-label study

*Abbreviations*: *ES-SCLC* extensive-stage small-cell lung cancer, *iv* intravenous, *LS-SCLC* limited-stage small-cell lung cancer, *Q2W* every 2 weeks, *Q3W* every 3 weeks

**Table 2:** Original data extracted from included studies

Study		Patients	Survival outcomes				Drug response							Grade 3-4 AEs %
			mOS (months)	mPFS (months)	OSR-1y	PFSR-6m	ORR	DCR	CR	PR	SD	mDOR (months)	Ongoing response	
2020	Charles [12]	Arm A (*n* = 228)	10.8 (9.2-12.9)	4.5 (4.3-5.4)	45.10%	34.20%	70.60%	88.20%	1.80%	68.90%	17.50%	4.2 (1.0-26.0)	NR	76.70%
		Arm B (*n* = 225)	9.7 (8.6-10.7)	4.3 (4.2-4.4)	39.60%	NR	61.80%	86.70%	0.90%	60.90%	24.90%	3.7 (1.4-25.8)	19.30%	74.90%
2017	Ott [13]	24	9.7 (4.1-NR)	1.9 (1.7-5.9)	37.70%	29.20%	33.30%	37.60%	4.20%	29.20%	4.20%	19.4 (3.6-20.0)	8.30%	8.30%
2018	Shirish [14]	45	9.6 (7.0-12.0)	1.4 (1.3-2.8)	37.00%	20.00%	11.10%	NR	2.20%	8.90%	NR	10.8 (5.8-NR)	NR	8.90%
2019	Kim [15]	26	9.1 (6.5–15.0)	5.0 (2.7–6.7)	40.70%	34.60%	23.10%	80.80%	3.90%	19.20%	57.70%	9.1 (3.6–11.6)	NR	34.60%
2019	Welsh [16]	33	8.4 (6.7-10.1)	6.1 (4.1–8.1)	29.20%	51.50%	13.16%	33%	3%	12%	18%	NR	NR	6.00%
2020	Welsh [17]	40	39.5 (8.0–71.0)	19.7 (8.8–30.5)	74.70%	80%	78.79%	97%	9.10%	69.70%	18.20%	NR	3.30%	15.00%

*Abbreviations*: *CR* complete response, *DCR* disease control rate, *Grade 3–4 AEs* grade 3–4 treatment-related adverse effects, *mOS* median overall survival, *mPFS* median progression-free survival, *mDOR* median duration of response, *NR* no relevant statistic data, *ORR* objective response rate, *OSR-1y* 1-year overall survival rate, *PFSR-6m* progression-free survival rate at 6 months, *PR* partial response, *SD* stable disease

### Antitumor efficacy

**Fig. 2:**
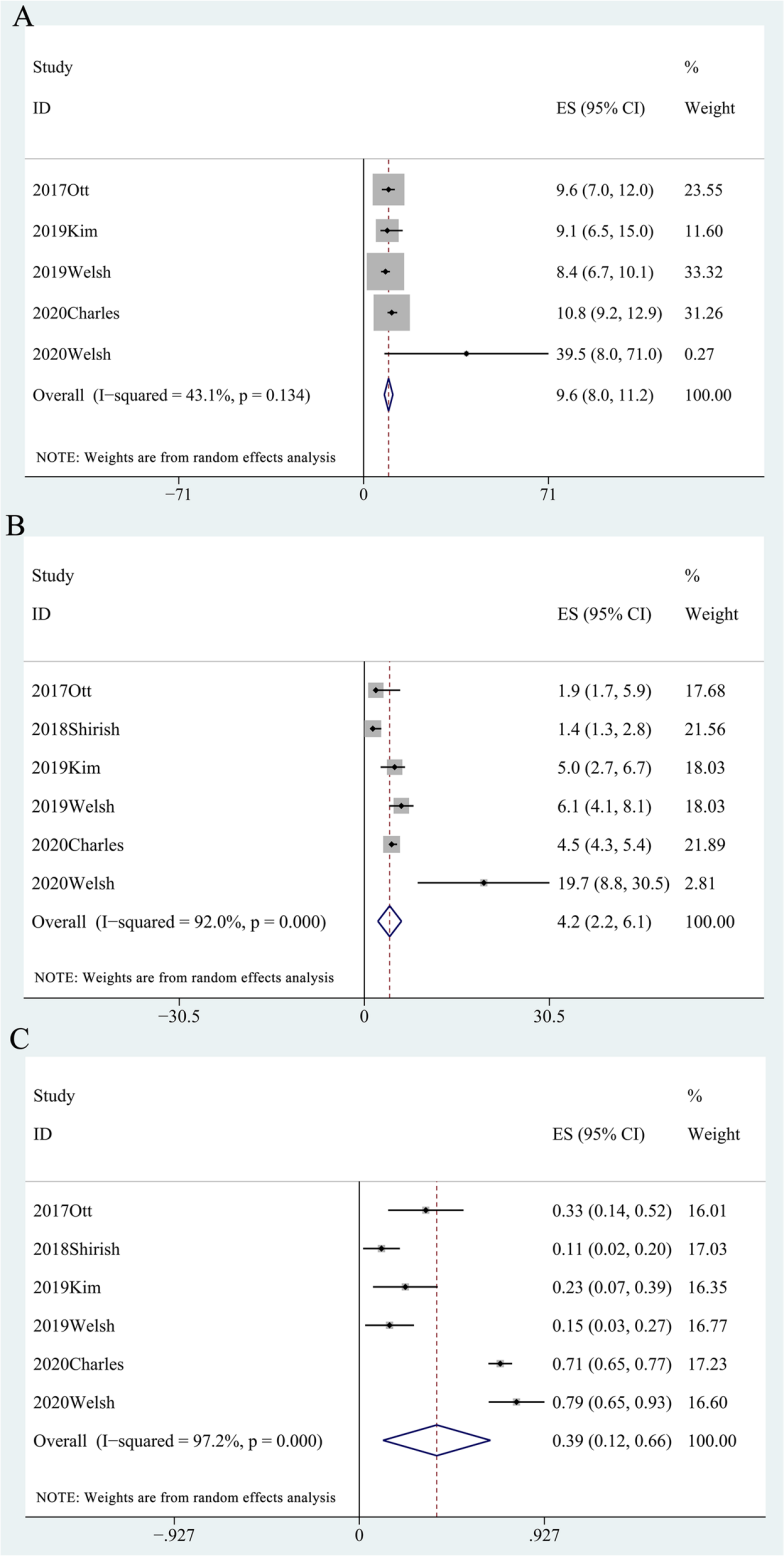
Forest plot of mOS **A**, mPFS **B**, and ORR **C**

**Fig. 3:**
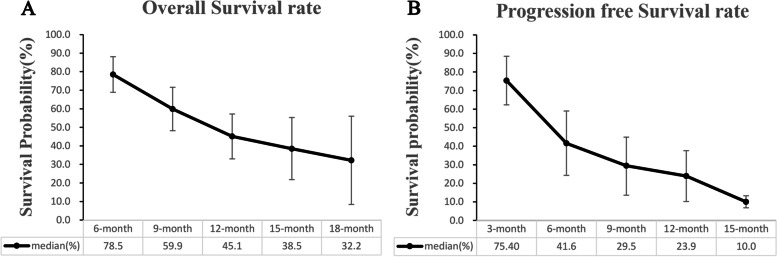
Tendency chart of OS rate (6-18 months, **A**) and PFS rate (3-15 months, **B**)

Some studies reported mOS and its range [12, 14–17] involving 377 patients; the pooled mOS was 9.6 months (95% CI, 8.0-11.2, *I*^2^ = 43.1%) (Table S4, Fig. 2A), with the 6-month overall survival rate (OSR-6m), OSR-9m, OSR-12m, OSR-15m, and OSR-18m being 78.50% (95% CI, 68.9-88.1%), 59.90% (95% CI, 48.2-71.6%), 45.10% (95% CI, 33.0-57.2%), 38.50% (95% CI, 21.8-55.3%), and 32.20% (95% CI, 8.4-56.0%) (Fig. 3), respectively.

Some studies reported mPFS and its range [12–17] involving 396 patients; the pooled mPFS was 4.2 months (95% CI, 2.2-6.1, *I*^2^ = 92.0%) (Table S5, Fig. 2B), with the progression-free survival rate at 3 months (PFSR-3m), PFSR-6m, PFSR-9m, PFSR-12m, and PFSR-15m being 75.4% (95% CI, 62.3-88.5%), 41.60% (95% CI, 24.3-59.0%), 29.5% (95% CI, 13.6-44.9%), 23.9% (95% CI, 10.2-37.6%), and 10.0% (95% CI, 6.8-13.3%) (Fig. 3), respectively.

In some studies reporting drug response, the pooled ORR [12–17] was 38.80% (95% CI, 11.9-65.67%, *I*^2^ = 97.2%) (Table S6, Fig. 2C); the pooled DCR [11, 12, 14–16] was 69.30% (95% CI, 51.6-87.0%) (Table S7), the pooled CR was 2.20% (95% CI, 0.8-3.7%), the pooled PR was 34.70% (95% CI, 7.8-61.5%), and the pooled SD was 20.90% (95% CI, 9.1-32.6%) (Table S8).

### Toxicity

We summarized the total AEs in all of the included studies (Table 3). The most common AEs were neutropenia (90.16%), dysphagia (41.96%), platelet count decrease (34.87%), anemia (53.21%), esophagitis (32.89%), alopecia (32.89%), fatigue (32.7%), nausea (32.51%), decreased appetite (29.39%), and decreased white blood cell count (26.3%).

**Table 3:** Total adverse effects in SCLC patients

Adverse events	Studies involved	Event/total	%
**Hematological system AEs**			
Neutropenia	2	229/254	90.16
Anemia	4	174/327	53.21
Platelet count decrease	2	91/261	34.87
WBC count decrease	4	91/346	26.30
Febrile neutropenia	2	7/66	10.61
**Digestive system AEs**			
Dysphagia	2	30/73	41.96
Esophagitis	2	25/73	32.89
Nausea	5	118/363	32.51
Constipation	4	83/332	25.00
Decreased appetite	1	67/228	29.39
Diarrhea	5	76/363	20.94
Vomiting	2	40/254	15.75
Increased aspartate transaminase level	1	6/45	13.33
Norexia	3	13/99	13.13
Abdominal pain	1	5/45	11.11
Stomatitis	1	1/26	3.85
Duodenitis	1	1/40	2.50
Pancreatitis	1	1/40	2.50
**Respiratory system AEs**			
Dyspnea	4	76/346	21.97
Pneumonia	3	53/294	18.03
Lung infection	1	5/40	12.50
Respiratory failure	1	1/40	2.50
**Skin AEs**			
Rash	6	51/396	12.88
Pruritus	5	46/372	12.37
Dry skin	1	2/24	8.33
Excessive tearing	1	2/24	8.33
Radiation dermatitis	2	6/73	8.22
**Circulative system AEs**			
Peripheral edema	2	22/268	8.21
Pericarditis	1	1/40	2.50
Sinus tachycardia	1	1/40	2.50
Flushing	1	1/40	2.50
**Nervous system AEs**			
Peripheral sensory neuropathy	2	17/66	25.76
Peripheral motor neuropathy	1	3/26	11.54
Neuropathy	1	5/45	11.11
**Motor system AEs**			
Myalgia	2	12/50	24.00
Arthralgia	2	10/64	15.63
Back pain	2	33/273	12.09
Arthritis	1	2/40	5.00
Muscle weakness	1	1/33	3.03
Chest wall pain	1	1/40	2.50
**Electrolyte disturbance AEs**			
Hyponatremia	3	32/294	10.88
Hypokalemia	1	3/40	7.50
**Urological system AEs**			
Creatinine increased	1	9/40	22.50
Chronic kidney disease	1	2/40	5.00
**Endocrine system AEs**			
Type I diabetes mellitus	1	3/26	11.54
Hypothyroidism	2	24/254	9.49
**Systemic AEs**			
Fatigue	5	121/370	32.70
Asthenia	3	58/278	20.86
Pyrexia	1	35/228	15.35
Generalized weakness	1	5/45	11.11
**Others AEs**			
Alopecia	1	75/228	32.89
Cough	4	71/346	20.52
Dizziness	3	48/229	16.05
Noncardiac chest pain	1	6/45	13.33
Headache	4	39/332	11.75
Insomnia	2	27/252	10.71
Pain	1	3/33	9.09
Confusion	1	1/40	2.50

*Abbreviations*: *AEs* adverse effects, *SCLC* small-cell lung cancer

**Fig. 4:**
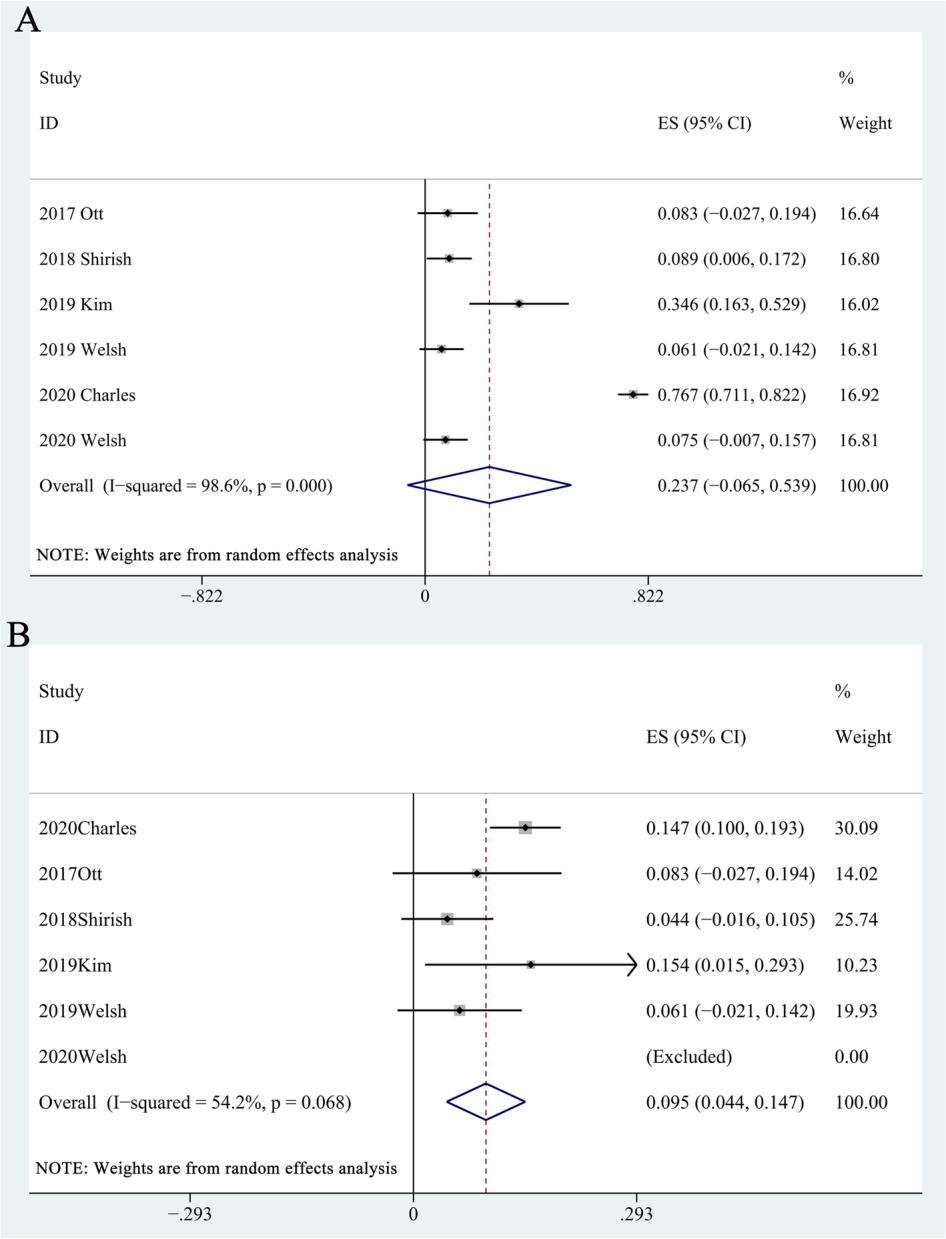
Forest plots of grade 3-4 adverse effects rate (**A**) and discontinuation treatment rate (**B**)

Some studies evaluated the grade 3-4 AEs% [12–17] involving 391 patients. We gathered all grade 3-4 AEs in the included studies (Table S9); the pooled grade 3-4 AE rate was 20.88% (95% CI, 1.22-54.85%, *I*^2^ = 98.6%) (Table S10, Fig. 4A). The severe AEs included neutropenia (36.68%), anemia (14.19%), pneumonitis (6.57%), acute coronary syndrome (4.44%), colitis/intestinal ischemia (4.17%), and respiratory failure (2.5%).

Some studies reported discontinuation treatment rate [12–17] involving 396 patients; the pooled discontinuation treatment rate was 9.50% (95% CI, 4.40-14.70%; *I*^2^ = 54.2%, *P* = 0.068) (Table S11, Fig. 4B).

### Subgroup analysis

To confirm whether the efficacy of pembrolizumab plus chemotherapy varied among different subgroups, all subgroup effects for the OS, PFS, and ORR were calculated in every category of the relevant variables. The results of most subgroups were consistent with the overall results.

Subgroup analysis indicated the antitumor activity of the combination of pembrolizumab and chemotherapy (Q2W, 200 mg for 16 cycles) was superior to chemotherapy alone in mOS, mPFS, and ORR. The patients with a median age ≤ 65 years old and with LS-SCLC also showed a better response. No transparent influence in study design, chemotherapy drug, or region was observed. Table 4 lists the results of the subgroup analyses.

**Table 4:** Subgroup analysis of mOS, mPFS, and ORR of Pembrolizumab combined with chemotherapy in SCLC

Subgroups	mOS				mPFS				ORR			
	No. of studies	ES (95% CI)	***P***	***I***^2^	No. of studies	ES (95% CI)	***P***	***I***^2^	No. of studies	ES (95% CI)	***P***	***I***^2^
**Total**												
**Study design**												
Noncomparative Open-label study	4	9.0 (7.1-10.9)	0.234	29.70%	5	4.3 (1.7-7.0)	0	89.00%	5	32.1% (7.3-56.9%)	0	94.20%
Randomized control study	1	10.8 (9.2-12.9)	-	-	1	4.5 (4.3-5.4)	NR	NR	1	-	-	-
**Chemotherapy drug**												
Platinum plus etoposide	4	9.7 (7.7-11.7)	0.072	57.10%	5	4.0 (1.8-6.3)	0	93.30%	5	41.9% (11.4-72.3%)	0	97.60%
Platinum plus etoposide and paclitaxel	1	9.1 (6.5–15.0)	-	-	1	5.0 (2.7–6.7)	NR	NR	1	23.10% (6.9-39.3%)	-	-
**Clinical setting**												
Q3w, 200 mg, more than 30 cycles	2	10.4 (8.9-11.9)	0.45	0.00%	2	3.0 (0.0-6.0)	0	97.70%	2	39.34% (0.22–93.33%)	0.082	60.00%
Q3w, 200 mg, 16cycles	2	19.8 (0-49.2)	0.053	73.20%	2	11.8 (1.3-22.3)	0.016	82.90%	2	46.26% (0.85-97.21%)	0	97.80%
Q2w, 20 mg/kg, 48 cycles	-	-	-	-	1	1.9 (1.7-5.9)	NR	NR	1	33.3% (14.5-52.2%)	-	-
Q3w, 175 mg/m^2^, 5 cycles	1	9.1 (6.5-15.0)	-	-	1	5.0 (2.7-6.7)	NR	NR	1	23.1% (6.9-39.3%)	-	-
**SCLC phase**												
ES-SCLC	4	9.5 (8.3-10.7)	0.315	15.40%	5	3.7 (1.9-5.6)	0	92.50%	5	30.8% (0.7-61.0%)	0	97.40%
LS-SCLC	1	39.5 (8.0–71.0)	-	-	1	19.7 (8.8-30.5)	-	NR	1	78.79% (64.84-92.74%)	-	-
**Study phase**												
I	1	8.4 (6.7-10.1)	-	-	2	4.0 (0.1-7.9)	-	-	2	22.8% (5.2-40.3%)	0.113	60.20%
II	2	9.5 (7.3-11.6)	0.842	0.00%	2	3.1 (0.4–5.8)	0.001	90.80%	2	15.2% (4.1-26.3%)	0.208	37.00%
III	1	10.8 (9.2-12.9)	-	-	1	4.5 (4.3-5.4)	-	-	1	70.6% (64.7-76.52)	-	-
I/II	1	39.5 (8.0-71.0)	-	-	1	19.7 (8.8-30.5)	-	-	1	78.79% (64.84-92.74%)	-	-
**Median age**												
≤ 65 years old	3	9.9 (6.8-12.9)	0.03	71.40%	4	4.9 (2.5-7.2)	0.131	56.20%	4	49.8% (20.0-79.5%)	0	96.20%
> 65 years old	2	9.5 (7.3-11.6)	0.842	0.00%	2	3.1 (0.4–5.8)	0.001	90.80%	2	15.2% (4.1-26.3%)	0.208	37.00%
**Region**												
America	4	9.7 (7.7-11.7)	0.072	57.10%	5	4.0 (1.8-6.3)	0	93.30%	5	41.9% (11.4-72.3%)	0	97.60%
Korea	1	9.1 (6.5–15.0)	-	-	1	5.0 (2.7–6.7)	NR	NR	1	23.10% (6.9-39.3%)	-	-
**Whether combine with radiation therapy**												
No	3	10.2 (8.8-11.6)	0.644	0.00%	4	3.2 (1.1-5.2)	0	93.60%	4	34.23% (6.94–69.30%)	0	97.70%
Yes	2	19.8 (0-49.2)	0.053	73.20%	2	11.8 (1.3–22.3)	0.016	82.90%	2	46.26% (0.085-97.21%)	0	97.80%

*Abbreviations*: *ES-SCLC* extensive-stage small cell lung cancer, *ES* effect size, *LS-SCLC* limited-stage small cell lung cancer, *ORR* objective response rate, *OS* overall response, *PFS* progression-free survival, *95% CI* 95% confidence interval

### Comparison with chemotherapy alone

Only one RCT compared pembrolizumab plus chemotherapy (etoposide and platinum [EP]) and chemotherapy alone (EP) [12]. The results showed that pembrolizumab plus chemotherapy remarkably advanced PFS (hazard ratio [HR] = 0.75; 95% CI, 0.61-0.91; *P* = 0.0023) and prolonged OS (HR = 0.80; 95% CI = 0.64-0.98; *P* = 0.0164). The ORR was 70.6% in the pembrolizumab plus chemotherapy group and 61.8% in the chemotherapy alone group, and the grade 3-4 AEs were 76.7 and 74.9%, respectively.

### Sensitivity analysis

Because high heterogeneity was found in mPFS, ORR, and grade 3-4 AEs, we evaluated the impact of every study on the pooled results to demonstrate stability and sensitivity. The results revealed that the outcomes of mOS (Figure S1A), mPFS (Figure S1B), ORR (Figure S1C), and grade 3–4 AEs (Figure S1D) were reliable and stable.

### Publication bias

The publication bias of the pooled mOS (Egger’s test: *P* = 0.388; Begg’s test: *P* = 0.806; Figure S2A) and pooled mPFS (Egger’s test: *P* = 0.925; Begg’s test: *P* = 0.851; Figure S2B) were examined with Egger’s and Begg’s regression tests. The results did not reveal any publication bias in mOS and mPFS.

## Discussion

SCLC is highly aggressive and associated with poor survival outcomes, and it lacks optional and effective treatment arms. Although SCLC is sensitive to conventional first-line chemotherapy drugs, most patients relapse [29]. The efficacy of pembrolizumab plus conventional chemotherapy remains controversial. This meta-analysis is the first to analyze the efficacy and risks of the combination of pembrolizumab and conventional chemotherapy in SCLC patients. Our results showed that pembrolizumab is a promising immunotherapy drug for combination with conventional chemotherapy drugs.

In the analysis of survival data, we found that the pooled mOS, pooled mPFS, and pooled ORR were 9.6 months with 95% CI, 8.0-11.2, 4.2 months with 95% CI, 2.2-6.1, and 38.8% with 95% CI, 11.9-65.67%, respectively. Charles et al. [12] compared pembrolizumab plus chemotherapy with chemotherapy alone and demonstrated that the combination of pembrolizumab plus chemotherapy can improve mOS (HR, 0.80; 95% CI, 0.64-0.98; *P* = 0.0164) and mPFS (HR, 0.75; 95% CI, 0.61-0.91; *P* = 0.0023) without unexpected toxicities. Baize et al. [29] evaluated the efficacy of first-line chemotherapy alone in 162 patients, and their results showed that the mOS and mPFS were 7.5 months and 4.7 months, respectively, and the ORR was 49% in SCLC. Chung et al. [8] evaluated the efficacy of pembrolizumab alone in 107 SCLC patients, and the results showed that the mPFS and mOS were 2.0 months and 9.1 months, respectively; the ORR was 18.7% in SCLC. As demonstrated by the above results, we propose that pembrolizumab combined with chemotherapy is superior to a single agent. Based on this finding, we conducted further analyses. Via the programmed cell death 1 (PD-1) signaling pathway, the release of negative regulators of immune activation (immune checkpoints) restricts antitumor responses and leads to unparalleled rates of persistent tumor responses in patients with multifarious cancers [30]. Pembrolizumab is a highly efficient monoclonal antibody with high selectivity that can directly handicap the correlation of PD-1 and PD-L1 or PD-L2 [31, 32]. However, the expression of PD-L1 and PD-L2 in SCLC is lower than that in non-small-cell lung cancer (NSCLC) [33]. Therefore, monotherapy with pembrolizumab has certain limitations [34]. Second, the emergence of drug resistance and decreased chemotherapy efficacy remains a clinical challenge [7]. Third, Melosky hypothesized that chemotherapy can be a potential regulator for PD-L1 expression, which may promote a tumor cell’s response to pembrolizumab [35]. A promising comprehensive treatment strategy for SCLC would be a significant advancement [36]. We propose that pembrolizumab plus chemotherapy may have better antitumor efficacy to prolong OS and PFS in SCLC than chemotherapy alone or pembrolizumab alone.

In subgroup analyses, age and SCLC phase were the main factors influencing the efficacy of pembrolizumab plus chemotherapy. Patients with a median age < 65 years old had better results than the subgroup of patients > 65 years old. Several factors may account for this finding. First, a higher tumor mutation burden (TMB) was used for prediction of better durable clinical benefit (DCB) of immune checkpoint inhibitors (ICIs) in younger patients [37]. The poorer response of older patients to ICIs may be related to immunosenescence characterized by decreased T cell proliferation, which is considered an age-related change in the immunity of the host, suggesting that patients with increased age had a worse response to ICI therapy that may influence the efficacy of ICIs in elderly people with a high prevalence of malignancies [38]. Finally, treatment-related AEs occur less often in younger patients than in older patients. Patients with LS-SCLC have better organ function reserve with a more sensitive drug response [17] and therefore a longer survival. As a PD1/PD-L1 inhibitor, PD1 expression level may be also the key factor of efficacy of Pembrolizumab plus chemotherapy [39]; besides, smoking may also influence the response to pembrolizumab [40]. Unfortunately, we have not obtained enough data to analyze the impact of these two factors. We will continue to follow up this research in the future.

We also analyzed the AEs of pembrolizumab combined with chemotherapy, including all grade AEs and grade 3-4 AEs. The most common AEs were neutropenia (90.16%), anemia (53.21%), dysphagia (41.96%), platelet count decrease (34.87%), esophagitis (32.89%), alopecia (32.89%), fatigue (32.7%), nausea (32.51%), decreased appetite (29.39%), and white blood cell count decrease (26.3%), which were manageable and similar to those reported in other solid tumor types, including SCLC [41]. Hematological system relevant AEs (including neutropenia, anemia, and platelet count decrease) and checkpoint inhibitor pneumonia (CIP) should be given attention. First- or second-line chemotherapy that damages bone marrow is the most common cause of leukopenia (chemotherapy-induced myelosuppression, CIM), which is more likely to occur in elderly patients and can be associated with decreased survival and tumor response [42], so control of CIM in the treatment of SCLC is crucial. CIP is a severe immune-related adverse event (irAE) that can complicate ICI therapy and is potentially life-threatening [40]. No definitive evidence has discerned the exact mechanism of CIP, but current evidence demonstrated that CIP is a lung disease with inflammatory infiltration due to autoantibody-mediated immunological processes with upregulated T cell activities, cytokine production levels, and amplification of complement [43]. CIP should be given more attention because of its considerable harm [44], although the incidence is low (18.03% in our study). In conclusion, pembrolizumab combined with chemotherapy is relatively safe in SCLC patients, but hematological system relevant AEs and CIP need to be treated carefully.

Several limitations should be considered when interpreting our results. First, although we included almost all of the up-to-date studies in this emerging field, only 6 studies with a relatively small number of eligible participants were included in our meta-analysis. Second, all the included documents were written in English, which may lead to language bias. Third, only one study analyzed the results of pembrolizumab plus chemotherapy compared with chemotherapy alone. Because almost all the included studies lacked control or therapy groups without available control group data, we subjectively assessed the efficacy and risk of selection bias without an exact direct statistical conclusion. We lacked sufficient data on PD-1/PD-L1 expression to analyze its correlation with the efficacy and risk of pembrolizumab. Fourth, although sensitive analysis demonstrated our outcomes of pooled mPFS, ORR, and grade 3-4 AEs were reliable and stable and subgroup analysis demonstrated there was no obvious impact on total pooled outcomes, the LS-SCLC, and second-line chemotherapy in our included studies may cause possible impact in outcome. Finally, the inhomogeneity between the level of participants and the level of the trial led to significant heterogeneity.

## Conclusion

In summary, pembrolizumab with conventional chemotherapy is a beneficial therapeutic schedule with assessable PFS, OS, ORR, and grade 3-4 AEs. Subgroup analyses indicated that patient age (< 65 years old) and SCLC phase (LS-SCLC) are the main factors affecting efficacy of pembrolizumab plus chemotherapy, but treatment-related hematological system AEs and CIP should be given attention during treatment. More high-quality RCTs with large samples are needed to further validate our conclusions.

## Supplementary Information

**Additional file 1: Figure S1.** Sensitivity analysis of mOS (A), mPFS (B), ORR (C) and grade 3-4 adverse effects (D).

**Additional file 2: Figure S2.** Publication bias of mOS (A) and mPFS (B).

**Additional file 3: Table S1.** PRISMA 2009 Checklist.

**Additional file 4: Table S2.** Search strategy.

**Additional file 5: Table S3.** GRADE Quality assessment by therapeutic strategy and study design for the outcomes of survival, and adverse events.

**Additional file 6: Table S4.** Pooled median overall survival in SCLC patients.

**Additional file 7: Table S5.** Pooled median progression-free survival in SCLC patients.

**Additional file 8: Table S6.** Pooled objective response rate in SCLC patients.

**Additional file 9: Table S7.** Pooled disease control rate in SCLC patients.

**Additional file 10: Table S8.** Confirmed efficacy results (investigator-assessed) in the total population.

**Additional file 11: Table S9.** Pooled discontinuation treatment rate in SCLC patients for included studies.

**Additional file 12: Table S10.** Pooled grade 3-4 adverse effects rate in SCLC patients.

**Additional file 13: Table S11.** Grade 3-4 adverse effects in included studies.

## Data Availability

The data sets used and/or analyzed during the current study are available from the corresponding author on reasonable request.

## Abbreviations

AEs: Adverse effects
CI: Confidence interval
CR: Complete response
CIP: Checkpoint inhibitor pneumonia
CIM: Chemotherapy-induced myelosuppression
DCB: Durable clinical benefit
DCR: Disease control rate
ES: Effects sizes
EP: Etoposide and platinum
ECOG status: Eastern Cooperative Oncology Group performance status
FDA: US Food and Drug Administration
HR: Hazard ratio
irAEs: Immune-related adverse event
ICI: Immune checkpoint inhibitors
LI: Lower interval
mOS: Median overall survival
mPFS: Median progression free survival
mDOR: Median duration of response
OSR-12mo: Overall survival rate at 12 months
OS: Overall survival
ORR: Objective response rate
PD-L1/PD-1: Programmed death ligand 1
PFS: Progression-free survival
PFSR-3m: Progression-free survival rate at 3 months
PR: Partial response
PRISMA: Preferred Reporting Items for Systematic Review and Meta-Analysis
RCT: Randomized control trial
TBM: Tumor mutation burden
SCLC: Small-cell lung cancer
SD: Stable disease
UL: Upper interval
