# Supplementary figures and images for: The benefits and risks of pembrolizumab in combination with chemotherapy as first-line therapy in small-cell lung cancer: a single-arm meta-analysis of noncomparative clinical studies and randomized control trials
Source: World J Surg Oncol. 2021 Oct 14;19:298. doi: 10.1186/s12957-021-02410-3 (PMC8515717; doi:10.1186/s12957-021-02410-3)

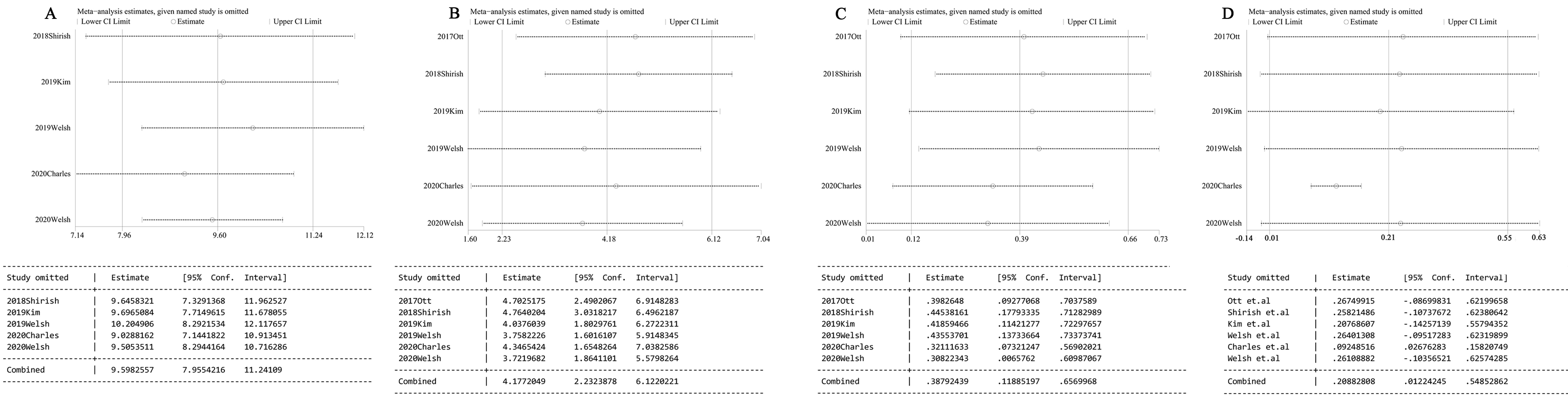

Supplement: Supplementary file 1 — Additional file 1: Figure S1. Sensitivity analysis of mOS (A), mPFS (B), ORR (C) and grade 3-4 adverse effects (D). [file 12957_2021_2410_MOESM1_ESM.tif]

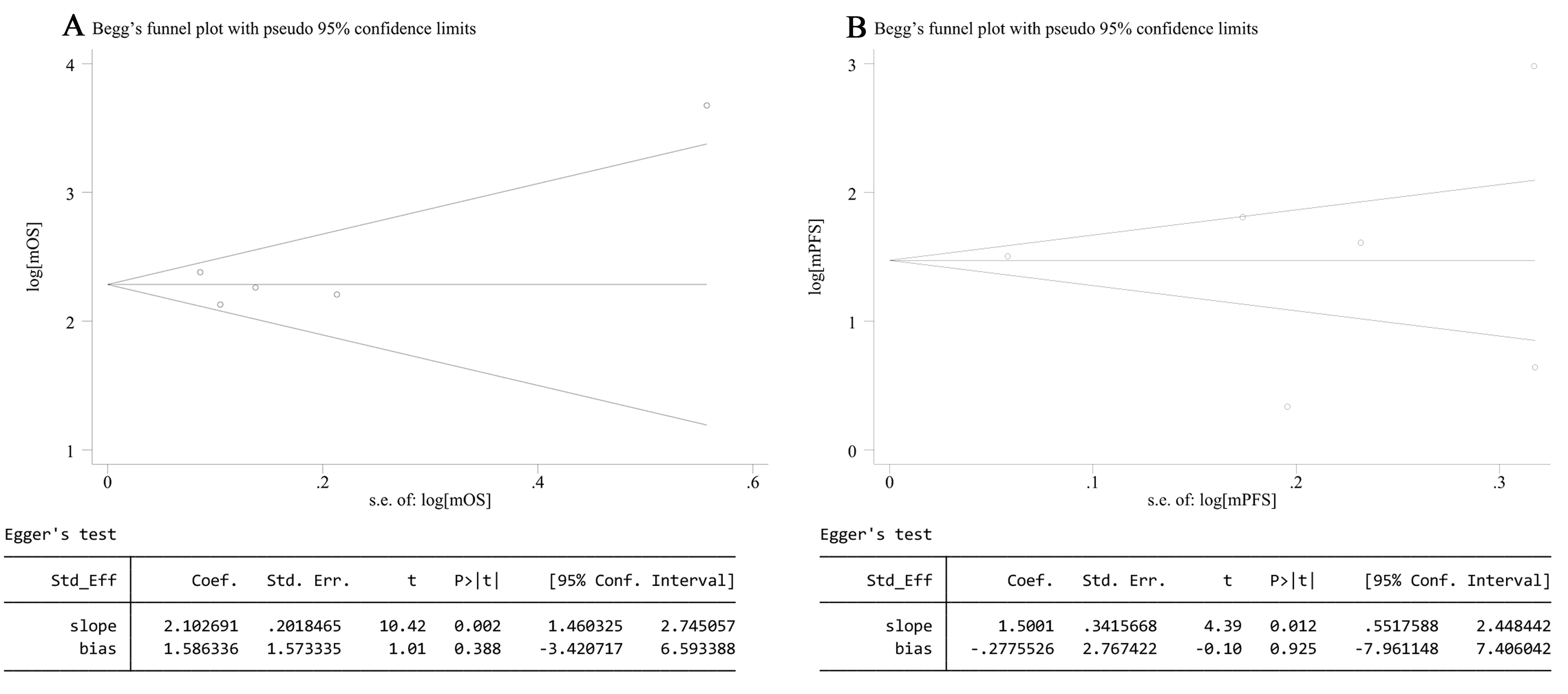

Supplement: Supplementary file 2 — Additional file 2: Figure S2. Publication bias of mOS (A) and mPFS (B). [file 12957_2021_2410_MOESM2_ESM.tif]
