# Supplementary material for: The benefits and risks of pembrolizumab in combination with chemotherapy as first-line therapy in small-cell lung cancer: a single-arm meta-analysis of noncomparative clinical studies and randomized control trials
Source: World J Surg Oncol. 2021 Oct 14;19:298. doi: 10.1186/s12957-021-02410-3 (PMC8515717; doi:10.1186/s12957-021-02410-3)
Supplement: Supplementary file 4 — Additional file 4: Table S2. Search strategy. [file 12957_2021_2410_MOESM4_ESM.docx]

**Table S2** Search strategy

| **PubMed**  The database was searched on December 20, 2020, n=320.  Search Strategy:  (Pembrolizumab[Title/Abstract] OR SCH-900475[Title/Abstract] OR Keytruda[Title/Abstract] OR MK-3475[Title/Abstract] OR Lambrolizumab[Title/Abstract]) AND (Chemotherapy[Title/Abstract]) AND (Small Cell Lung Carcinoma[Title/Abstract] OR Small Cell Lung Cancer[Title/Abstract] OR Oat Cell Lung Cancer[Title/Abstract] OR Small Cell Cancer Of The Lung[Title/Abstract] OR Carcinoma, Small Cell Lung[Title/Abstract] OR Oat Cell Carcinoma of Lung[Title/Abstract]) |
| --- |
| **Web of Science**  The database was searched December 20, 2020, n=334.  Search Strategy:  1 TOPIC:(“Pembrolizumab” OR “SCH-900475” OR “Keytruda” OR “MK-3475” OR “Lambrolizumab”)(9270)  2 TOPIC: (“chemotherapy”) (315861)  3 TOPIC: (“Small cell lung cancer” OR “Small Cell Lung Cancer” OR “Oat Cell Lung Cancer” OR “Small Cell Lung Carcinoma” OR “Carcinoma, Small Cell Lung” OR “Oat Cell Carcinoma Of Lung”) (46554)  4 #1 AND #2 AND #3 (880) |
| **EMBASE**  The database was searched on December 20, 2020, n=786.  Search Strategy:  ('Pembrolizumab':ti,ab,kw OR 'SCH-900475':ti,ab,kw OR 'Keytruda':ti,ab,kw OR 'Keytruda':ti,ab,kw OR 'MK-4375':ti,ab,kw OR 'Lambrolizumab':ti,ab,kw) AND ('Chemotherapy':ti,ab,kw) AND ('Small Cell Lung Carcinoma':ti,ab,kw OR 'Small Cell Lung cancer':ti,ab,kw OR 'Oat Cell Lung cancer':ti,ab,kw OR 'Small Cell Cancer Of Lung':ti,ab,kw OR 'Carcinoma, Small Cell Lung':ti,ab,kw OR 'Oat Cell Carcinoma Of Lung':ti,ab,kw) |
| **Cochrane Library**  The database was searched December 20, 2020, n=254.  Search Strategy:  (“Pembrolizumab” OR “SCH-900475” OR “Keytruda” OR “MK-4375” OR “Lambrolizumab”): ti,ab,kw AND (“Chemotherapy”): ti,ab,kw AND (“Small Cell Lung Carcinoma” OR “Small Cell Lung cancer” OR “Oat Cell Lung cancer” OR “Small Cell Cancer Of Lung” OR “Carcinoma, Small Cell Lung” OR “Oat Cell Carcinoma Of Lung”)(Word variations have been searched) |
| **Ovid MEDLINE**  The database was searched on December 20, 2020, n=11.  Search Strategy:  1 Pembrolizumab.ab. (4046)  2 SCH-900475.ab. (1)  3 Keytruda.ab. (73)  4 MK-3475.ab. (46)  5 Lambrolizumab.ab. (13)  6 or/1-5 [ Pembrolizumab] (4125)  7 Chemotherapy.ab. (7488)  8 Small Cell Lung Carcinoma.ab. (2245)  9 Small Cell Lung Cancer.ab. (1434)  10 Oat Cell Lung Cancer.ab. (2864)  11 Small Cell Cancer Of The Lung.ab. (21823)  12 Carcinoma, Small Cell Lung.ab. (1615)  13 Oat Cell Carcinoma of Lung.ab. (2245)  14 or/8-13 [ Small Cell Lung Carcinoma] (24511)  15 6 and 7 and 14 (11) |
| **ScienceDirect**  The database was searched on December 20, 2020, n=111.  Search Strategy:  Title, abstract, keywords: ((“Pembrolizumab” OR “SCH-900475” OR “Keytruda” OR “MK-4375” OR “Lambrolizumab”) and (“Chemotherapy”) and (“Small Cell Lung Carcinoma” OR “Small Cell Lung cancer” OR “Oat Cell Lung cancer” OR “Small Cell Cancer Of Lung” OR “Carcinoma, Small Cell Lung” OR “Oat Cell Carcinoma Of Lung”)) |
| **Scopus**  The database was searched on December 20, 2020, n=1157.  Search Strategy:  TITLE-ABS-KEY ((“Pembrolizumab” OR “SCH-900475” OR “Keytruda” OR “MK-4375” OR “Lambrolizumab”) and (“Chemotherapy”) and (“Small Cell Lung Carcinoma” OR “Small Cell Lung cancer” OR “Oat Cell Lung cancer” OR “Small Cell Cancer Of Lung” OR “Carcinoma, Small Cell Lung” OR “Oat Cell Carcinoma Of Lung”)) |

**Note:** The combined text and medical subject heading (MeSH) terms used were: “Pembrolizumab”, “Chemotherapy” and “Small cell lung cancer”.
