# Supplementary material for: The benefits and risks of pembrolizumab in combination with chemotherapy as first-line therapy in small-cell lung cancer: a single-arm meta-analysis of noncomparative clinical studies and randomized control trials
Source: World J Surg Oncol. 2021 Oct 14;19:298. doi: 10.1186/s12957-021-02410-3 (PMC8515717; doi:10.1186/s12957-021-02410-3)
Supplement: Supplementary file 5 — Additional file 5: Table S3. GRADE Quality assessment by therapeutic strategy and study design for the outcomes of survival, and adverse events. [file 12957_2021_2410_MOESM5_ESM.docx]

**Table S3** GRADE Quality assessment by therapeutic strategy and study design for the outcomes of survival, and adverse events.

| **Primary outcomes** | **No. of Studies** | **No. of participants** | **Difference（95%CI）** | **Quality assessment** | | | | | **Quality** |
| --- | --- | --- | --- | --- | --- | --- | --- | --- | --- |
|  |  |  |  | **Risk^a^ of bias** | **Inconsistency** | **Indirectness** | **Imprecision** | **Publication bias^b^** |  |
| **ORR%** | 6 | 389 | 38.80% (11.9%-65.67%) | Low | No inconsistency | No indirectness | No imprecision | Likely (-1) | Moderate |
| **DCR%** | 5 | 274/344 | 69.30% (51.6%-87.0%) | Low | No inconsistency | No indirectness | No imprecision | Likely (-1) | Moderate |
| **CR%** | 6 | 11/389 | 2.2% (0.8%-3.7%) | Low | No inconsistency | No indirectness | No imprecision | Unlikely | High |
| **PR%** | 6 | 200/389 | 34.70% (7.8%-61.5%) | Low | No inconsistency | No indirectness | No imprecision | Unlikely | High |
| **SD%** | 5 | 68/344 | 20.90% (9.1%-32.6%) | Low | No inconsistency | No indirectness | No imprecision | Unlikely | High |
| **Survival** | | | | | | | | | |
| mOS months | 5 | 372 | 9.6 (8.0-11.2) | Low | No inconsistency | No indirectness | No imprecision | Unlikely | High |
| **OSR** | | | | | | | | | |
| 6-month | 6 | 306/396 | 78.50% (68.9%-88.1%) | Low | No inconsistency | No indirectness | No imprecision | Unlikely | High |
| 9-month | 6 | 233/396 | 59.90% (48.2%-71.6%) | Low | No inconsistency | No indirectness | No imprecision | Unlikely | High |
| 12-month | 6 | 179/396 | 45.10% (33.0%-57.2%) | Low | No inconsistency | No indirectness | No imprecision | Unlikely | High |
| 15-month | 5 | 140/343 | 38.50% (21.8%-55.3%) | Low | No inconsistency | No indirectness | No imprecision | Unlikely | High |
| 18-month | 4 | 97/337 | 32.20% (8.4%-56.0%) | Low | No inconsistency | No indirectness | No imprecision | Unlikely | High |
| mPFS months | 6 | 396 | 4.2 (2.2-6.1) | Low | No inconsistency | No indirectness | No imprecision | Unlikely | High |
| **PFSR** | | | | | | | | | |
| 3-month | 6 | 315/396 | 75.4% (62.3%-88.5%) | Low | No inconsistency | No indirectness | No imprecision | Unlikely | High |
| 6-month | 6 | 152/396 | 41.60% (24.3%-59.0%) | Low | No inconsistency | No indirectness | No imprecision | Unlikely | High |
| 9-month | 6 | 98/396 | 29.5% (13.6%-44.9%) | Low | No inconsistency | No indirectness | No imprecision | Unlikely | High |
| 12-month | 6 | 79/396 | 23.9% (10.2%-37.6%) | Low | No inconsistency | No indirectness | No imprecision | Unlikely | High |
| 15-month | 5 | 34/356 | 10.0% (6.8%-13.3%) | Low | No inconsistency | No indirectness | No imprecision | Unlikely | High |
| **mDOR months** | 3 | 278 | 8.6 (4.8-12.5) | Low | No inconsistency | No indirectness | Serious (-1) | Unlikely | Moderate |
| **Grade 3-4 AEs %** | 6 | 190/391 | 23.70% (-6.5%-53.9%) | Low | No inconsistency | No indirectness | No imprecision | Unlikely | High |
| Neutropenia | 3 | 106/289 | 36.68% (31.12%-42.23%) | Low | No inconsistency | No indirectness | No imprecision | Unlikely | High |
| Anemia | 3 | 41/289 | 14.19% (10.16%-18.21%) | Low | No inconsistency | No indirectness | No imprecision | Unlikely | High |
| Thrombocytopenia | 1 | 31/223 | 13.9% (9.36%-18.44%) | Low | No inconsistency | No indirectness | No imprecision | Unlikely | High |
| Leukopenia | 2 | 27/263 | 10.27% (6.60%-13.93%) | Low | No inconsistency | No indirectness | No imprecision | Unlikely | High |
| Febrile neutropenia | 2 | 5/66 | 7.58% (1.19%-13.96%) | Low | No inconsistency | No indirectness | No imprecision | Unlikely | High |
| Lung infection | 1 | 3/40 | 7.5% (2%-21%) | Low | No inconsistency | No indirectness | No imprecision | Unlikely | High |
| Hyponatremia | 3 | 8/111 | 7.21% (2.40%-12.02%) | Low | No inconsistency | No indirectness | No imprecision | Unlikely | High |
| Pneumonia | 3 | 19/289 | 6.57% (3.71%-9.43%) | Low | No inconsistency | No indirectness | No imprecision | Unlikely | High |
| Hypokalemia | 1 | 2/40 | 5% (1%-17%) | Low | No inconsistency | No indirectness | No imprecision | Unlikely | High |
| Acute coronary syndrome | 1 | 2/45 | 4.44% (1-15%) | Low | No inconsistency | No indirectness | No imprecision | Unlikely | High |
| Type I diabetes mellitus | 2 | 3/71 | 4.23% (1%-12%) | Low | No inconsistency | No indirectness | No imprecision | Unlikely | High |
| Colitis/intestinal ischemia | 1 | 1/24 | 4.17% (0-21%) | Low | No inconsistency | No indirectness | No imprecision | Unlikely | High |
| Bilirubin elevation | 1 | 1/24 | 4.17% (0-21%) | Low | No inconsistency | No indirectness | No imprecision | Unlikely | High |
| Asthenia | 3 | 11/282 | 3.9% (1.64%-6.16%) | Low | No inconsistency | No indirectness | No imprecision | Unlikely | High |
| Myalgia | 1 | 1/26 | 3.47% (0-20%) | Low | No inconsistency | No indirectness | No imprecision | Unlikely | High |
| Autoimmune disorder | 1 | 1/33 | 3.03% (0-15%) | Low | No inconsistency | No indirectness | No imprecision | Unlikely | High |
| Paresthesia | 1 | 1/33 | 3.03% (0-15%) | Low | No inconsistency | No indirectness | No imprecision | Unlikely | High |
| Fatigue | 2 | 7/263 | 2.66% (0.72%-4.61%) | Low | No inconsistency | No indirectness | No imprecision | Unlikely | High |
| Diarrhea | 2 | 7/263 | 2.66% (0.72%-4.61%) | Low | No inconsistency | No indirectness | No imprecision | Unlikely | High |
| Duodenitis | 1 | 1/40 | 2.5% (0-13%) | Low | No inconsistency | No indirectness | No imprecision | Unlikely | High |
| Pancreatitis | 1 | 1/40 | 2.5% (0-13%) | Low | No inconsistency | No indirectness | No imprecision | Unlikely | High |
| Respiratory failure | 1 | 1/40 | 2.5% (0-13%) | Low | No inconsistency | No indirectness | No imprecision | Unlikely | High |
| Pruritus | 1 | 1/40 | 2.5% (0-13%) | Low | No inconsistency | No indirectness | No imprecision | Unlikely | High |
| Flushing | 1 | 1/40 | 2.5% (0-13%) | Low | No inconsistency | No indirectness | No imprecision | Unlikely | High |
| Confusion | 1 | 1/40 | 2.5% (0-13%) | Low | No inconsistency | No indirectness | No imprecision | Unlikely | High |
| Pericarditis | 1 | 1/40 | 2.5% (0-13%) | Low | No inconsistency | No indirectness | No imprecision | Unlikely | High |
| Sinus tachycardia | 1 | 1/40 | 2.5% (0-13%) | Low | No inconsistency | No indirectness | No imprecision | Unlikely | High |
| Chest wall pain | 1 | 1/40 | 2.5% (0-13%) | Low | No inconsistency | No indirectness | No imprecision | Unlikely | High |
| Chronic kidney disease | 1 | 1/40 | 2.5% (0-13%) | Low | No inconsistency | No indirectness | No imprecision | Unlikely | High |
| Dyspnea | 2 | 5/263 | 1.9% (0.25%-3.55%) | Low | No inconsistency | No indirectness | No imprecision | Unlikely | High |
| Rash | 3 | 4/296 | 1.35% (0.04%-2.67%) | Low | No inconsistency | No indirectness | No imprecision | Unlikely | High |
| Vomiting | 1 | 2/223 | 0.9% (0-2.1%) | Low | No inconsistency | No indirectness | No imprecision | Unlikely | High |
| Back pain | 1 | 1/223 | 0.45% (0-1.3%) | Low | No inconsistency | No indirectness | No imprecision | Unlikely | High |
| Cough | 1 | 1/223 | 0.45% (0-1.3%) | Low | No inconsistency | No indirectness | No imprecision | Unlikely | High |
| Pyrexia | 1 | 1/223 | 0.45% (0-1.3%) | Low | No inconsistency | No indirectness | No imprecision | Unlikely | High |
| Nausea | 1 | 1/223 | 0.45% (0-1.3%) | Low | No inconsistency | No indirectness | No imprecision | Unlikely | High |
| Decreased appetite | 1 | 1/223 | 0.45% (0-1.3%) | Low | No inconsistency | No indirectness | No imprecision | Unlikely | High |
| Constipation | 1 | 1/223 | 0.45% (0-1.3%) | Low | No inconsistency | No indirectness | No imprecision | Unlikely | High |

**Abbreviations:** ORR: Objective response rate; DCR: disease control rate; CR: complete response; PR: partial response; SD: stable disease; OS: overall survival; OSR: overall survival rate; mOS months: median overall survival months; PFSR: progression-free survival rate; Grade 3-4 AEs: Grade 3-4 adverse event rate. mDOR: median duration of response

^a^ Risk of bias assessed using the Newcastle-Ottawa Scale (NOS) for non-randomized studies.

^b^ Publication bias was assessed by Egger’s and Begg’s tests.
