# Supplementary material for: The benefits and risks of pembrolizumab in combination with chemotherapy as first-line therapy in small-cell lung cancer: a single-arm meta-analysis of noncomparative clinical studies and randomized control trials
Source: World J Surg Oncol. 2021 Oct 14;19:298. doi: 10.1186/s12957-021-02410-3 (PMC8515717; doi:10.1186/s12957-021-02410-3)
Supplement: Supplementary file 6 — Additional file 6: Table S4. Pooled median overall survival in SCLC patients. [file 12957_2021_2410_MOESM6_ESM.docx]

**Table S4** Pooled median overall survival in SCLC patients.

| **Study** | | **OS** | | **Weight** |
| --- | --- | --- | --- | --- |
|  |  | **Median** | **95%CI** |  |
| Total | | 9.6 | (8.0-11.2) | 100.00% |
| 2017 | Ott et al | 9.7 | (4.1-NR) | NR |
| 2018 | Shirish et al | 9.6 | (7.0-12.0) | 23.50% |
| 2019 | Kim et al | 9.1 | (6.5–15.0) | 11.60% |
| 2019 | Welsh et al | 8.4 | (6.7-10.1) | 33.32% |
| 2020 | Charles et al | 10.8 | (9.2-12.9) | 31.26% |
| 2020 | Welsh et al | 39.5 | (8.0‒71.0) | 0.27% |
| Overall (*I*^2^ = 43.1%, P = 0.134); Egger's test(P = 0.388) | | | | |

**Abbreviations:** OS: overall survival; 95%CI: 95% confidence interval.
