# Supplementary material for: The benefits and risks of pembrolizumab in combination with chemotherapy as first-line therapy in small-cell lung cancer: a single-arm meta-analysis of noncomparative clinical studies and randomized control trials
Source: World J Surg Oncol. 2021 Oct 14;19:298. doi: 10.1186/s12957-021-02410-3 (PMC8515717; doi:10.1186/s12957-021-02410-3)
Supplement: Supplementary file 7 — Additional file 7: Table S5. Pooled median progression-free survival in SCLC patients. [file 12957_2021_2410_MOESM7_ESM.docx]

**Table S5** Pooled median progression-free survival in SCLC patients.

| **Study** | | **mPFS** | | **Weight** |
| --- | --- | --- | --- | --- |
|  |  | **Median** | **95%CI** |  |
| Total | | 4.2 | (2.2-6.1) | 100.00% |
| 2017 | Ott et al | 1.9 | (1.7-5.9) | 17.68% |
| 2018 | Shirish et al | 1.4 | (1.3-2.8) | 21.56% |
| 2019 | Kim et al | 5.0 | (2.7–6.7) | 18.03% |
| 2019 | Welsh et al | 6.1 | (4.1–8.1) | 18.03% |
| 2020 | Charles et al | 4.5 | (4.3-5.4) | 21.89% |
| 2020 | Welsh et al | 19.7 | (8.8‒30.5) | 2.81% |
| Overall (*I*^2^ = 92.0%, P = 0.000); Egger's test(P = 0.925) | | | | |

**Abbreviations:** mPFS: median progression-free survival; 95%CI: 95% confidence interval.
