# Supplementary material for: The benefits and risks of pembrolizumab in combination with chemotherapy as first-line therapy in small-cell lung cancer: a single-arm meta-analysis of noncomparative clinical studies and randomized control trials
Source: World J Surg Oncol. 2021 Oct 14;19:298. doi: 10.1186/s12957-021-02410-3 (PMC8515717; doi:10.1186/s12957-021-02410-3)
Supplement: Supplementary file 8 — Additional file 8: Table S6. Pooled objective response rate in SCLC patients. [file 12957_2021_2410_MOESM8_ESM.docx]

**Table S6** Pooled objective response rate in SCLC patients.

| **Study** | | **ORR** | | **Weight** |
| --- | --- | --- | --- | --- |
|  |  | **Median** | **95%CI** |  |
| Total | | 38.80% | 11.9%-65.67% | 100.00% |
| 2017 | Ott et al | 33.30% | 14.5%-52.2% | 16.00% |
| 2018 | Shirish et al | 11.10% | 1.9%-20.3% | 17.00% |
| 2019 | Kim et al | 23.10% | 6.9%–39.3% | 16.33% |
| 2019 | Welsh et al | 15.20% | 2.9%-27.4% | 16.88% |
| 2020 | Charles et al | 70.60% | 64.70%-76.52% | 17.20% |
| 2020 | Welsh et al | 78.79% | 64.84%-92.74% | 16.58% |
| Overall (*I*^2^ = 97.2%, P = 0.000); Egger's test(P = 0.018) | | | | |

**Abbreviations:** ORR: objective response rate; SCLC: small cell lung cancer.
