# Supplementary material for: The benefits and risks of pembrolizumab in combination with chemotherapy as first-line therapy in small-cell lung cancer: a single-arm meta-analysis of noncomparative clinical studies and randomized control trials
Source: World J Surg Oncol. 2021 Oct 14;19:298. doi: 10.1186/s12957-021-02410-3 (PMC8515717; doi:10.1186/s12957-021-02410-3)
Supplement: Supplementary file 9 — Additional file 9: Table S7. Pooled disease control rate in SCLC patients. [file 12957_2021_2410_MOESM9_ESM.docx]

**Table S7** Pooled disease control rate in SCLC patients.

| **Study** | | **DCR** | | **Weight** |
| --- | --- | --- | --- | --- |
|  |  | **Median** | **95%CI** |  |
| Total | | 69.30% | 51.6%-87.0% | 100.00% |
| 2017 | Ott et al | 37.60% | 18.1%-56.9% | 17.70% |
| 2019 | Kim et al | 80.80% | 65.6%–95.9% | 19.28% |
| 2019 | Welsh et al | 33.00% | 17.2%-49.4% | 18.94% |
| 2020 | Charles et al | 88.20% | 84.0%-92.4% | 22.17% |
| 2020 | Welsh et al | 97.00% | 91.1%-99.4% | 21.97% |
| Overall (*I*^2^ = 95.0%, P = 0.000); Egger's test(P = 0.098) | | | | |

**Abbreviations:** DCR: disease control rate; NR: no relevant statistic data.
