# Supplementary material for: The benefits and risks of pembrolizumab in combination with chemotherapy as first-line therapy in small-cell lung cancer: a single-arm meta-analysis of noncomparative clinical studies and randomized control trials
Source: World J Surg Oncol. 2021 Oct 14;19:298. doi: 10.1186/s12957-021-02410-3 (PMC8515717; doi:10.1186/s12957-021-02410-3)
Supplement: Supplementary file 10 — Additional file 10: Table S8. Confirmed efficacy results (investigator-assessed) in the total population. [file 12957_2021_2410_MOESM10_ESM.docx]

**Table S8** Confirmed efficacy results (investigator-assessed) in the total population.

| **Efficacy** | **Studies involved** | **Pooled** | **95%CI** | **P** | ***I*^2^** |
| --- | --- | --- | --- | --- | --- |
| CR | 6 | 2.20% | 0.8%-3.7% | 0.752 | 0.00% |
| PR | 6 | 34.70% | 7.8%-61.5% | 0.000 | 97.30% |
| SD | 6 | 20.90% | 9.1%-32.6% | 0.000 | 85.50% |
| ORR | 6 | 38.80% | 11.9%-65.67% | 0.000 | 97.20% |
| DCR | 5 | 69.30% | 51.6%-87.0% | 0.000 | 95.00% |
| 1-y OSR | 6 | 45.10% | 33.0%-57.2% | 0.000 | 79.30% |
| 6-mo PFSR | 6 | 41.60% | 24.3%-59.0% | 0.000 | 91.40% |

**Abbreviations:** CR: complete response rate; DCR: disease control rate; ORR: objective response rate; OSR-1y: overall survival at 1year rate; PFSR-6m: progression free at 6 months rate; PR: partial response rate; SD: stable disease rate; 95%CI: 95% confidence interval.
