# Supplementary material for: The benefits and risks of pembrolizumab in combination with chemotherapy as first-line therapy in small-cell lung cancer: a single-arm meta-analysis of noncomparative clinical studies and randomized control trials
Source: World J Surg Oncol. 2021 Oct 14;19:298. doi: 10.1186/s12957-021-02410-3 (PMC8515717; doi:10.1186/s12957-021-02410-3)
Supplement: Supplementary file 11 — Additional file 11: Table S9. Pooled discontinuation treatment rate in SCLC patients for included studies. [file 12957_2021_2410_MOESM11_ESM.docx]

**Table 9** Pooled discontinuation treatment rate in SCLC patients for included studies

| **Study** | | **Discontinuation treatment rate** | | **Weight** |
| --- | --- | --- | --- | --- |
|  |  | **Median** | **95%CI** |  |
|  | Total | 9.50% | 4.40%-14.70% | 100% |
| 2020 | Charles et al | 14.70% | 10.00%-19.30% | 30.09% |
| 2017 | Ott et al | 8.33% | -2.70%-19.4% | 14.02% |
| 2018 | Shirish et al | 4.44% | -1.6%-10.5% | 25.74% |
| 2019 | Kim et al | 15.40% | 1.50%-29.30% | 10.23% |
| 2019 | Welsh et al | 6.06% | -2.1%-14.2% | 19.93% |
| 2020 | Welsh et al | 0.00% | - | - |
| Overall (I^2^=54.2%, P = 0.068) | | | | |

**Abbreviations:** 95%CI: 95% confidence interval
