# Supplementary material for: The benefits and risks of pembrolizumab in combination with chemotherapy as first-line therapy in small-cell lung cancer: a single-arm meta-analysis of noncomparative clinical studies and randomized control trials
Source: World J Surg Oncol. 2021 Oct 14;19:298. doi: 10.1186/s12957-021-02410-3 (PMC8515717; doi:10.1186/s12957-021-02410-3)
Supplement: Supplementary file 12 — Additional file 12: Table S10. Pooled grade 3-4 adverse effects rate in SCLC patients. [file 12957_2021_2410_MOESM12_ESM.docx]

**Table S10** Pooled grade 3-4 adverse effects in SCLC patients.

| **Study** | | **Grade 3-4 AEs** | | **Weight** |
| --- | --- | --- | --- | --- |
|  |  | **Median** | **95%CI** |  |
| Total | | 23.70% | -6.5%-53.9% | 100% |
| 2017 | Ott et al | 8.33% | -2.72%-19.39% | 16.64% |
| 2018 | Shirish et al | 8.90% | 0.57%-17.20% | 16.80% |
| 2019 | Kim et al | 34.62% | 16.33%-52.90% | 16.02% |
| 2019 | Welsh et al | 6.06% | -2.08%-14.20% | 16.81% |
| 2020 | Charles et al | 76.68% | 71.13%-82.23% | 16.92% |
| 2020 | Welsh et al | 7.50% | -0.66%-15.66% | 16.81% |
| Overall (*I*^2^ = 98.6%; P = 0.000); Egger's test(P = 0.457) | | | | |

**Abbreviations:** Grade 3-4 AEs: grade 3-4 adverse effects; 95%CI: 95% confidence interval.
