# Supplementary material for: The benefits and risks of pembrolizumab in combination with chemotherapy as first-line therapy in small-cell lung cancer: a single-arm meta-analysis of noncomparative clinical studies and randomized control trials
Source: World J Surg Oncol. 2021 Oct 14;19:298. doi: 10.1186/s12957-021-02410-3 (PMC8515717; doi:10.1186/s12957-021-02410-3)
Supplement: Supplementary file 13 — Additional file 13: Table S11. Grade 3-4 adverse effects in included studies. [file 12957_2021_2410_MOESM13_ESM.docx]

**Table S11** Grade 3-4 adverse effects in included studies.

| **Adverse events** | **Studies involved** | **Event/total** | **%** |
| --- | --- | --- | --- |
| **Hematological system AEs** | | | |
| Neutropenia | 3 | 106/289 | 36.68 |
| Anemia | 3 | 41/289 | 14.19 |
| Thrombocytopenia | 1 | 31/223 | 13.9 |
| Leukopenia | 2 | 27/263 | 10.27 |
| Febrile neutropenia | 2 | 5/66 | 7.58 |
| **Circulative system AEs** | | | |
| Acute coronary syndrome | 1 | 2/45 | 4.44 |
| Pericarditis | 1 | 1/40 | 2.5 |
| Sinus tachycardia | 1 | 1/40 | 2.5 |
| **Electrolyte disturbance AEs** | | | |
| Hyponatremia | 3 | 8/111 | 7.21 |
| Hypokalemia | 1 | 2/40 | 5 |
| **Digestive system AEs** | | | |
| Colitis/intestinal ischemia | 1 | 1/24 | 4.17 |
| Bilirubin elevation | 1 | 1/24 | 4.17 |
| Diarrhea | 2 | 7/263 | 2.66 |
| Duodenitis | 1 | 1/40 | 2.5 |
| Pancreatitis | 1 | 1/40 | 2.5 |
| Vomiting | 1 | 2/223 | 0.9 |
| Nausea | 1 | 1/223 | 0.45 |
| Decreased appetite | 1 | 1/223 | 0.45 |
| Constipation | 1 | 1/223 | 0.45 |
| **Respiratory system AEs** | | | |
| Lung infection | 1 | 3/40 | 7.5 |
| Pneumonia | 3 | 19/289 | 6.57 |
| Respiratory failure | 1 | 1/40 | 2.5 |
| Dyspnea | 2 | 5/263 | 1.9 |
| **Motor system AEs** | | | |
| Myalgia | 1 | 1/26 | 3.47 |
| Chest wall pain | 1 | 1/40 | 2.5 |
| Back pain | 1 | 1/223 | 0.9 |
| **Skin AEs** | | | |
| Pruritus | 1 | 1/40 | 2.5 |
| Rash | 3 | 4/296 | 1.35 |
| **Endocrine system AEs** | | | |
| Type I diabetes mellitus | 2 | 3/71 | 4.23 |
| **Nervous system AEs** | | | |
| Paresthesia | 1 | 1/33 | 3.03 |
| **Urological system AEs** | | | |
| Chronic kidney disease | 1 | 1/40 | 2.5 |
| **Others AEs** | | | |
| Asthenia | 3 | 11/282 | 3.9 |
| Autoimmune disorder | 1 | 1/33 | 3.03 |
| Fatigue | 2 | 7/263 | 2.66 |
| Flushing | 1 | 1/40 | 2.5 |
| Confusion | 1 | 1/40 | 2.5 |
| Cough | 1 | 1/223 | 0.9 |
| Pyrexia | 1 | 1/223 | 0.9 |

**Abbreviations:** AEs: adverse effects; SCLC: Small-cell lung cancer.
